# Impact of Aggregation on the Immunogenicity of Therapeutic Recombinant Biological Products

**DOI:** 10.3390/ph19101604

**Published:** 2026-10-09

**Authors:** Xiaoying Chen, Sha Guo, Xiaorui Feng, Zhenhao Zhou, Junkai Liu, Maoqin Duan, Yalan Yang, Pengfei He, Hao Wu

**Affiliations:** 1 School of Pharmaceutical Engineering, Shenyang Pharmaceutical University, Shenyang 110016, China; chenxiaoying@stu.syphu.edu.cn (X.C.); fengxiaorui@stu.syphu.edu.cn (X.F.); zhouzhenhao@stu.syphu.edu.cn (Z.Z.); liujunkai@stu.syphu.edu.cn (J.L.); 2 WHO Collaborating Centre for Standardization and Evaluation of Biologicals, State Key Laboratory of Drug Regulatory Science, NHC Key Laboratory of Research on Quality and Standardization of Biotech Products, NMPA Key Laboratory for Quality Research and Evaluation of Biological Products, National Institutes for Drug Control, Beijing 102629, China; guosha@nidc.org.cn (S.G.); duanmaoqin@nidc.org.cn (M.D.); yangyalan@nidc.org.cn (Y.Y.)

**Keywords:** protein aggregation, immunogenicity, therapeutic recombinant biologics, anti-drug antibodies, quality control

## Abstract

**Background**: Therapeutic recombinant biological products are essential in modern medicine, but their immunogenicity remains a major concern for both clinical safety and therapeutic efficacy. Protein aggregation may occur during manufacturing, storage, transportation, and administration, potentially increasing immune recognition. Here, we have reviewed the mechanisms underlying aggregation-associated immunogenicity and summarized strategies for mitigating the associated risks. **Methods**: We integrated published evidence on therapeutic recombinant proteins, monoclonal antibodies, antibody–drug conjugates, peptide and glycoprotein hormones, and recombinant blood products. We focused on aggregation-induced structural changes, immune activation pathways, differences among product types and aggregation states, and approaches for controlling aggregation and mitigating immunogenicity. **Results**: Protein aggregation creates or reveals neoepitopes by disrupting the native conformational state, exposing previously buried hydrophobic regions, and generating nonnative interfaces. Aggregates may further promote innate and adaptive immune responses through repetitive epitope presentation, pattern-recognition receptor signaling, complement activation, cytokine release, dendritic-cell maturation, enhanced antigen presentation, and B-cell receptor cross-linking, ultimately increasing the likelihood of anti-drug antibody formation. Aggregate size and morphology, protein characteristics, formulation, storage conditions, administration route, and other biological factors influence the immunogenic potential of aggregates. Thus, effective risk reduction requires integrated control strategies, including molecular and structural optimization, formulation design, manufacturing-process control, appropriate storage and transportation, and sensitive analytical monitoring. Emerging artificial-intelligence approaches may further facilitate the prediction and design of low-immunogenicity biologics. **Conclusions**: Protein aggregation is a multifactorial determinant of immunogenicity. Controlling aggregation throughout the product lifecycle is essential to reduce immune-mediated risks and maintain safety, stability, and efficacy of therapeutic recombinant biological products.

## 1. Introduction

Major breakthroughs in recombinant DNA technology in the 1970s have led to considerable advancements in the development and application of therapeutic recombinant biologics. With the widespread clinical use of these drugs, their immunogenicity has increasingly been recognized as a critical factor that affects both therapeutic efficacy and patient safety. Protein aggregation is a primary driver of enhanced immunogenicity. According to Rojekar et al. [1], aggregation can result in the formation of both soluble and insoluble aggregates, the latter of which are prone to precipitation. Insoluble aggregates typically manifest as either fibrillar or amorphous structures, depending on the intrinsic properties of the protein and its surrounding environment. Fibrillar aggregates are highly ordered, β-sheet-rich assemblies that are often amyloid in nature, whereas amorphous aggregates are disordered and lack defined higher-order structure. Both forms threaten protein stability and can compromise therapeutic efficacy.

**Figure 1:**
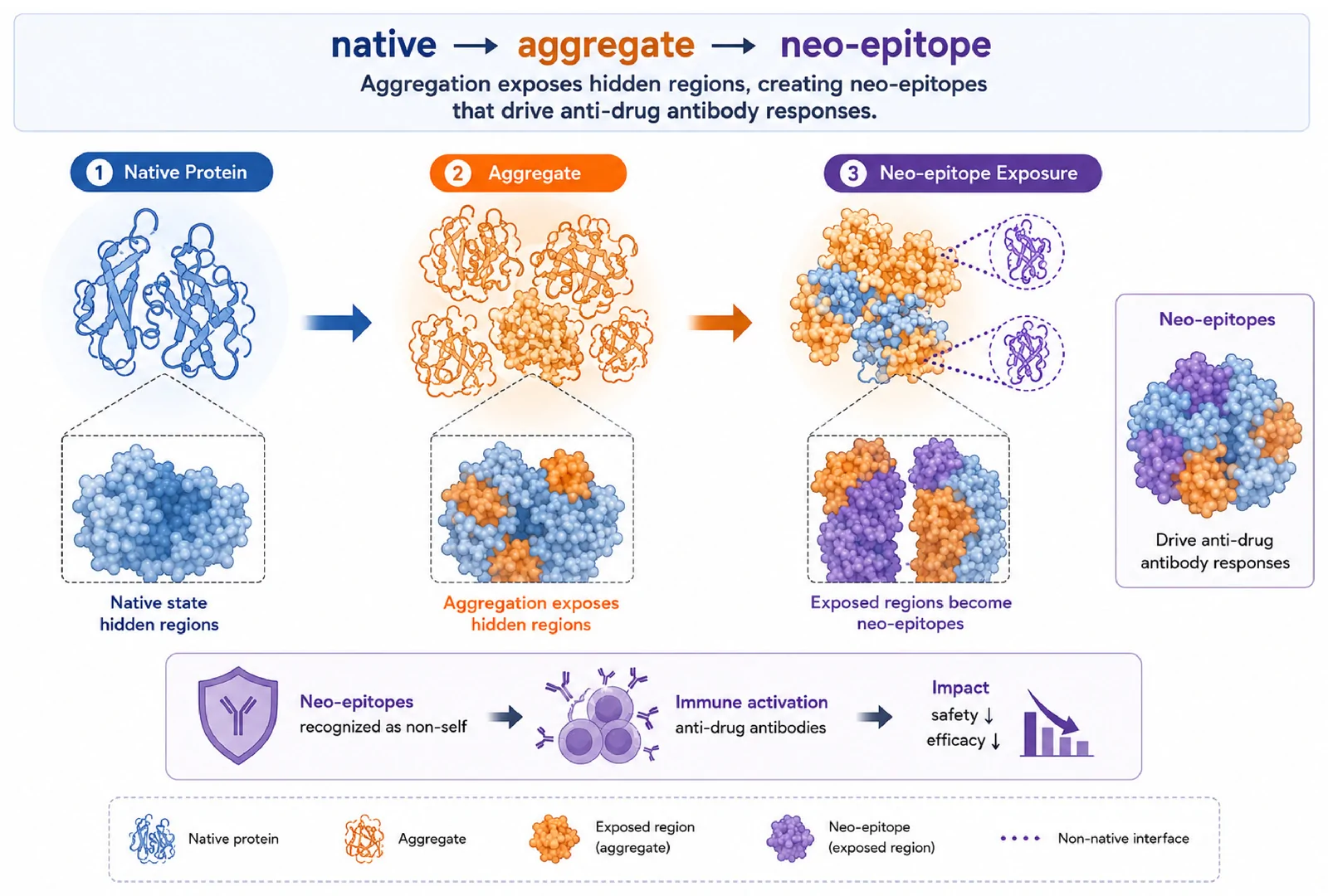
From native protein to aggregate-associated neoepitope exposure. Protein misfolding and aggregation may disrupt the native conformational state, expose previously buried structural regions, and generate nonnative interfaces. These changes may create or reveal neoepitopes, which are recognized by the immune system as nonself, potentially triggering anti-drug antibody responses and compromising the safety and efficacy of therapeutic proteins. In this schematic, blue areas represent the native protein, orange areas indicate aggregates or exposed regions, and purple areas highlight neo-epitopes. The arrows illustrate the process flow and its downstream consequences.

As illustrated in Figure 1, due to protein misfolding, unfolding-induced exposure of previously buried hydrophobic regions, or the formation of nonnative covalent/noncovalent interfaces, aggregated drug molecules may expose neoepitopes. These structures, which are immunologically “sequestered” in their native state, may activate the immune system and trigger the production of anti-drug antibodies (ADAs) [2]. This diminishes efficacy and may also lead to severe immune-mediated adverse reactions. Therefore, it is of paramount importance to investigate the mechanisms by which protein aggregation influences immunogenicity and explore strategies to reduce such responses for enhancing the safety and efficacy of therapeutic recombinant biologics.

Despite extensive reviews of the fundamental principles linking protein aggregation to enhanced immunogenicity, several gaps remain. First, previous reports have largely focused on protein therapeutics as a broad category, but the immunogenicity implications of aggregation vary considerably across different product classes. This review provides a comprehensive cross-product-class synthesis, explicitly comparing the immunogenicity characteristics of monoclonal antibodies (mAbs), antibody–drug conjugates (ADCs), peptide and glycoprotein hormone therapeutics, recombinant blood products, and biosimilars. Second, unlike prior reports, which have predominantly emphasized drug-related determinants (e.g., aggregate size, formulation excipients), this review integrates drug-related, patient-related, and stress-related determinants into a single, unified framework. Specifically, we examine how patient-specific factors—including physiological state, genetic background (e.g., HLA polymorphisms), and concomitant therapies—modulate immunogenicity risk in concert with drug-quality attributes and environmental stresses encountered during manufacturing, storage, and administration. Third, this review incorporates a forward-looking perspective on the role of artificial intelligence (AI) in mitigating aggregation-associated immunogenicity. Specifically, we discuss how deep-learning algorithms, generative AI, and protein co-evolution models can accelerate the prediction and rational design of low-immunogenicity biologics. Finally, unlike previous reports, here we address the immunogenicity across the full product lifecycle, including comparative analysis across different shelf life stages and administration routes (intravenous [IV], subcutaneous [SC], intravitreal, and pulmonary). Taken together, these contributions position this review as a comprehensive, clinically oriented resource that bridges mechanistic understanding with practical risk-mitigation strategies for the diverse landscape of therapeutic recombinant biological products.

## 2. Immunogenicity Characteristics of Different Types of Therapeutic Recombinant Biological Products

### 2.1. mAbs

As a cornerstone of therapeutic recombinant biologics, mAbs play a pivotal role in the treatment of cancers, autoimmune disorders, and other diseases. The immunogenicity of mAb therapeutics primarily stems from differences between their amino-acid sequences and those of endogenous human antibodies; even fully human antibodies cannot entirely escape such a risk. Early murine antibodies retain non-human sequences throughout their structure; chimeric antibodies retain murine sequences only in the variable regions, whereas their constant regions are human-derived. The non-human sequences can be directly recognized by the immune system as foreign antigens, thereby eliciting an ADA response [3]. For example, rituximab—an mAb targeting CD20, which is widely used in the treatment of non-Hodgkin lymphoma—consists of approximately 35% murine and 65% human sequences. ADA formation has been documented in some patients during treatment with rituximab, leading to reduced therapeutic efficacy [3].

Even in fully human antibodies, although the Fc region is matched to the human sequence, the complementarity-determining regions remain structurally unique and may present novel epitopes that T cells or B cells perceive as “nonself,” thereby triggering anti-idiotypic antibody responses [4]. Moreover, engineering modifications—such as site-directed mutations, or conjugation with toxins or radionuclides—or chemical alterations introduced during production—such as incorporation of nonnatural amino acids or aberrant glycosylation, can generate neoepitopes, further increasing the immunogenic risk [5,6,7].

For ADC products, the immunogenicity risks are multifactorial and involve both payload-specific and antibody-derived mechanisms. Despite the hapten function of cyto-toxic payloads, clinical data demonstrate that the immunogenicity profile is actually product-specific [8]. Namely, for several vc-MMAE-containing ADCs, the majority of ADA responses target the mAb domain rather than the payload, indicating that antibody-derived sequences—potentially altered by conjugation—may contribute substantially to immunogenicity. The cytotoxic payload can be taken up by antigen-presenting cells (APCs) through nonspecific endocytosis or receptor-mediated internalization, leading to its intracellular accumulation. High local concentrations of the payload may exert direct cytotoxic effects on dendritic cells (DCs) and macrophages, thereby potentially impairing their antigen-presenting functions. Furthermore, infusion-related reactions and cytokine-release syndrome (CRS) represent clinically relevant risks, often arising from rapid payload release and subsequent immune-cell activation. Antibody aggregation is generally irreversible, particularly when aggregates consist of numerous nonnative molecules. These aggregates can elicit immunogenicity in patients receiving mAb therapy via T-cell-dependent pathways and cytokine-mediated activation. Larger aggregates exhibit stronger immunogenic potential, and the response is further modulated by factors such as glycosylation patterns and the origin of aggregate products. Ultimately, immunogenic reactions can significantly diminish in vivo therapeutic effects and may precipitate type-I hypersensitivity reactions. Because immunogenicity affects both efficacy and safety, minimizing unnecessary immunogenicity is a critical consideration throughout the development and manufacturing of therapeutic recombinant biologics.

### 2.2. ADCs

ADCs are composed of a mAb covalently linked to a cytotoxic drug via a chemical linker. Both the linker and the drug are typically small, chemically synthesized molecules that act as exogenous haptens or neoepitopes to the immune system. Even when the antibody component is fully humanized, the chemical structure of the linker—such as valine–citrulline dipeptides or maleimide moieties—and nonnatural amino-acid residues introduced at conjugation sites may be directly recognized by the immune system. More critically, cytotoxic payloads—such as DM1 (a maytansinoid derivative) or MMAE (auristatin derivative)—can function as haptens, forming complete antigens upon conjugation to the antibody carrier, thereby eliciting ADA responses. Additionally, the conjugation process itself may induce random modifications in the antibody’s constant or variable regions, thereby disrupting native tolerance epitopes and further amplifying the immunogenic risk.

When ADCs aggregate, the resulting structures ADC aggregates display highly repetitive multivalent arrays of both drug-linker and antibody epitopes. These aggregates efficiently cross-link B-cell receptors and activate the complement system while being preferentially internalized and presented by pattern-recognition receptors (PRRs) on dendritic cells (DCs). Even more importantly, ADC aggregates may enter APCs via nonspecific endocytosis or receptor-mediated uptake, thereby releasing high concentrations of cytotoxic payload intracellularly. This can not only enhances the immunogenicity but also and directly damages DCs or macrophages, thereby disrupting normal antigen presentation and potentially precipitating severe adverse events such as infusion reactions and CRS. Considering the rapid expansion of the ADC market expanding rapidly, understanding the immunogenicity of these agents is critical for drug development, regulatory approval, and clinical use.

A prime example is trastuzumab emtansine (T-DM1), which couples humanized IgG1 trastuzumab to the DM1 payload via a non-cleavable linker. This modification signifi-cantly reduces colloidal and conformational stability, making T-DM1 more prone to aggregation relative to unconjugated trastuzumab. Carrasco-Triguero et al. [8] evaluated the immunogenicity of eight vc-MMAE-containing ADCs across 11 clinical trials, and reported posttreatment ADA incidence rates between 0% and 35.8%. Notably, the majority of treatment-induced ADAs were directed against the mAb domain of the ADCs (86–100%), suggesting a limited role of hapten-like structures in generating immune responses against these ADCs. Furthermore, the overall immunogenicity risks of these ADCs were comparable to those of conventional mAbs, indicating that the conjugation of hydrophobic payloads does not universally increase the immunogenicity risk. However, payload-specific effects cannot be entirely excluded, given that different ADCs employ distinct linkers and cytotoxic agents, and the immunogenicity profile may vary depending on the specific molecular construct.

Overall, ADCs display a higher immunogenicity risk than traditional mAbs. The risk stems not only from the haptenic nature of the small-molecule payload but also from the amplification of antibody immunogenicity through hydrophobic toxin-induced aggregation. Even when the antibody component is humanized, its conjugation with a hydrophobic drug lowers the aggregation threshold, thereby facilitating the formation of subvisible or soluble aggregates in clinical use. Through B-cell receptor cross-linking, complement activation, and efficient presentation of newly exposed epitopes, these aggregates ultimately induce ADA responses at a higher frequency than naked antibodies.

### 2.3. Peptide and Glycoprotein Hormone Therapeutics

Peptide and glycoprotein hormone therapeutics refer to biologics produced via recombinant DNA technology that possess hormone-like physiological regulatory functions. These agents mimic the structure and function of endogenous human hormones (e.g., insulin or erythropoietin [EPO]) and play critical roles in metabolic regulation, hematopoiesis, and endocrine homeostasis. As a key branch of therapeutic recombinant proteins, these molecules are relatively small, but often carry complex posttranslational modifications, such as glycosylation. Although such modifications contribute to their remarkable efficacy, they also introduce structural instability, increasing the risk of aggregation during production and storage, which in turn can trigger immunogenicity.

**Figure 2:**
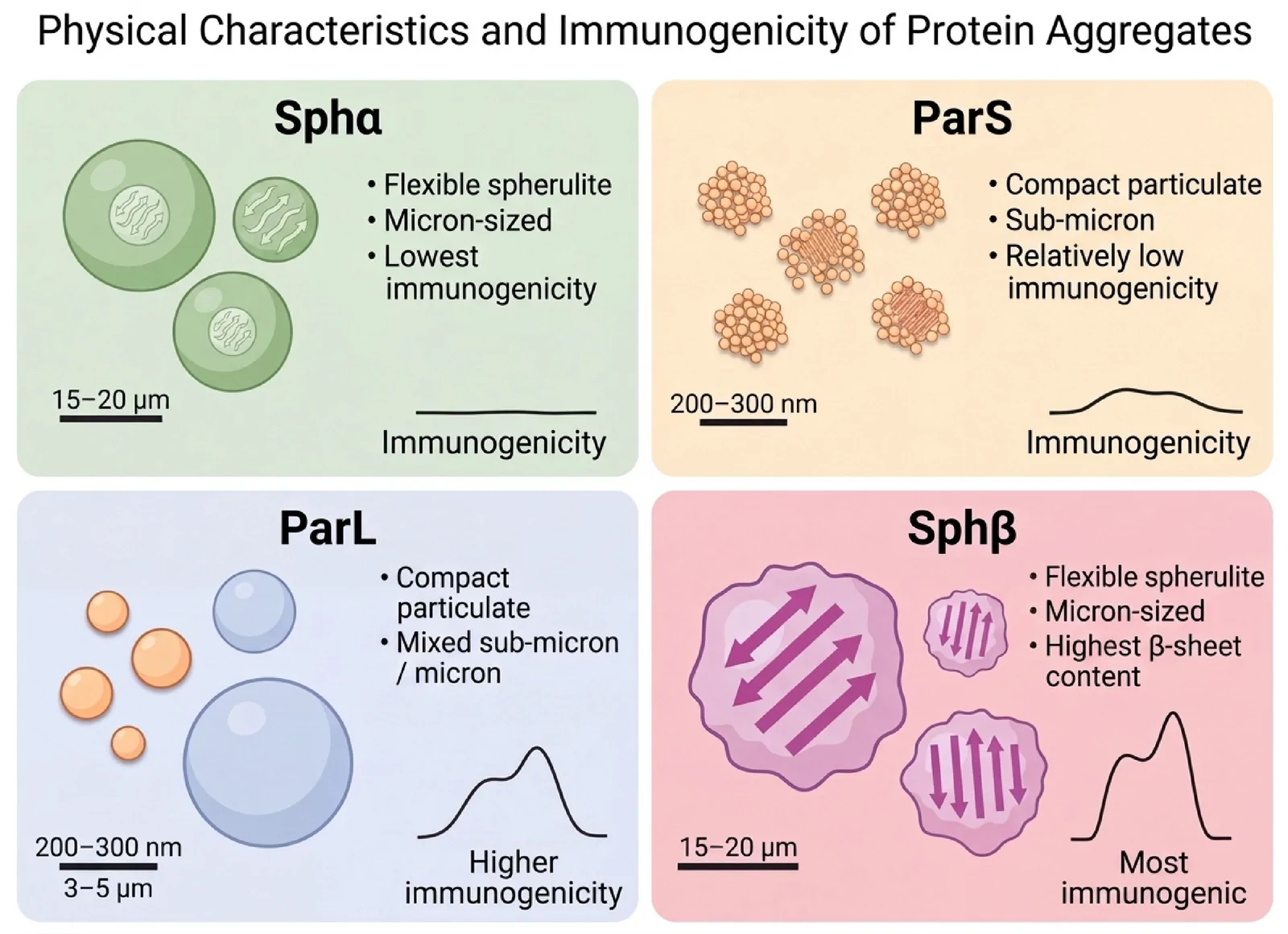
Ranking of the immunogenic potential of insulin aggregates. Sphα aggregates are micron-sized spherical particles (15–20 μm) with fibril-like-material internal protein arrangements, exhibiting the lowest immunogenicity. ParS aggregates consist of nano-sized particles (approximately 200–300 nm) featuring a native-like secondary structure (β/α-ratio ~0.90) and demonstrate relatively low immunogenicity. ParL aggregates are a mixture of nano-sized particles (200–300 nm) and micron-sized particulates (3–5 μm) with compact internal structures, which results in high immunogenicity. Sphβ aggregates, also micron-sized (15–20 μm), possess highly altered internal β-sheet structures (β/α-ratio ~1.86) and elicit the highest immunogenicity. By comparing the size, internal conformational order, and immune response intensity of these aggregates, this figure reveals the close correlation between the physicochemical properties of protein aggregates and their immunogenicity, providing a structural–functional basis for assessing the immunological risks associated with aggregates in biopharmaceutical products. In this schematic, the blue background highlights ParL aggregates, which consist of orange nano-sized particles and light blue micron-sized particulates. The pink background indicates Sphβ aggregates, where the purple double-headed arrows represent the aligned β-sheet structures.

Recombinant human insulin (rh-insulin) is a cornerstone therapy for diabetes. However, some patients experience reduced therapeutic efficacy due to the development of ADA during treatment [9]. Thorlaksen et al. [10] demonstrated varying immunogenic profiles of insulin aggregates formed under different pH and solvent conditions. Flexible aggregates with highly altered secondary structures—such as β-sheet-rich spherulites—showed high immunogenicity both in vitro and in vivo, whereas compact aggregates retaining near-native conformations—such as particulate structures—primarily exhibited immunogenicity in vivo. As shown in Figure 2, in vitro experiments revealed that flexible spherulites (hereafter abbreviated as Sph, with β-sheet-rich variants designated as Sphβ and less β-sheet-rich variants designated as Sphα) and compact particulate aggregates (hereafter abbreviated as Par, with larger-sized variants designated as ParL and smaller-sized variants designated as ParS) elicited differential immune responses. Namely, Sphβ and ParL showed stronger responses, whereas Sphα and ParS showed lower immunogenicity. In vivo, ParL and Sphβ aggregates induced ADA responses significantly higher than other aggregate forms or non-aggregated rh-insulin. Beyond these insulin-specific findings, the general relationship between aggregate size, structural order, and immunogenic potential is summarized in Table 1.

The intrinsic immunogenicity of peptide and glycoprotein hormone therapeutics primarily stems from their nature as exogenous biological macromolecules, which display subtle structural differences relative to endogenous human proteins. These differences include incomplete amino-acid sequence homology; critical posttranslational modifications, such as glycosylation patterns that differ from those of endogenous human EPO; and chemical alterations occurring during production or storage, such as deamidation or oxidation of insulin. Even in monomeric form, these structural features can render the molecules recognizable by the immune system as “nonself” antigens, directly triggering immune responses through T-cell-dependent pathways.

In a seminal study conducted in 1980, Wayne V. Moore and Paula Leppert demonstrated a substantial influence of aggregation of human growth hormone (hGH) preparations on antibody formation and persistence of immune responses in patients [11]. Through long-term observation of children with GH deficiency, they found that formulations containing a higher proportion of aggregated hGH were more likely to induce persistent antibodies with low affinity but high capacity. In rare cases of high-capacity antibody responses, not only did these persistent antibodies reduce drug efficacy, but they also led to a significant decline in patient growth rates.

The immunogenicity of recombinant human EPO (rhEPO) offers a landmark example of how protein aggregation can lead to severe clinical consequences. Casadevall et al. [12] reported 13 cases of pure red-cell aplasia in patients with chronic renal failure receiving epoetin therapy, demonstrating that the anemia was mediated by neutralizing anti-EPO antibodies. Serum from these patients completely blocked erythroid-colony formation by normal bone marrow cells; this inhibition was reversed by exogenous epoetin. Epidemiological investigations have linked the anemic outbreak with a formulation change introduced in 1998 for Eprex^®^ (epoetin alfa), in which human serum albumin, previously used as a stabilizer, was replaced with polysorbate 80 and glycine. Such a modification reduced the colloidal and conformational stability of the product, thereby increasing its propensity to form aggregates during storage and handling. However, it should be acknowledged that epidemiological associations alone cannot establish causality, as the PRCA outbreak coincided with other formulation, manufacturing, and batch-related changes; confounding factors, including possible batch-to-batch variability and production-related factors, cannot be excluded. Moreover, polysorbate 80 may promote the release of organic leachables from uncoated rubber stoppers in pre-filled syringes, and these leachables can act as adjuvants to further break immune tolerance. Critically, not only did the neutralizing antibodies (NAbs) recognize the exogenous therapeutic protein, but they also cross-reacted with endogenous EPO, thereby leading to severe transfusion-dependent anemia. This case powerfully illustrates that even minor formulation adjustments can profoundly alter the immunogenicity profile of a therapeutic recombinant protein by enhancing aggregation, and it remains a milestone warning for quality control and change management in the manufacturing of biologics.

### 2.4. Recombinant Blood Products

Recombinant blood products are bioactive therapeutics produced through gene recombination technologies, such as recombinant DNA techniques, by directed expression in in vitro cell systems, including Chinese hamster ovary (CHO) cells or yeast. These products are designed to replace traditional blood-derived therapeutics, such as albumin or coagulation factors, which are isolated from human plasma. By eliminating dependence on human plasma, recombinant blood products substantially reduce the risk of blood-borne pathogen transmission and overcome production limitations imposed by scarce plasma resources, thereby enabling large-scale, standardized industrial manufacturing. They represent a core direction in the blood product industry toward high-tech, high-safety, and environmentally sustainable production [13].

Recombinant human coagulation factors are critical therapeutics for the management of hemophilia. However, some patients experience reduced drug efficacy during treatment due to the development of ADA. For example, recombinant human coagulation factor VIII (rFVIII)—used in hemophilia A—primarily elicits immunogenicity in the form of NAbs targeting factor VIII [14]. Not only do these antibodies compromise hemostatic efficacy, but they can also provoke severe immune reactions, significantly impacting therapeutic outcomes. Therefore, it is essential to perform stringent control over product quality and manufacturing processes.

The intrinsic immunogenicity of recombinant blood products primarily arises from molecular differences between the exogenous therapeutic proteins and corresponding endogenous human proteins, but also from potential impurities introduced during production. Although recombinant technologies aim to mimic natural proteins, host cells—such as CHO or Pichia pastoris—may introduce non-human posttranslational modifications (e.g., aberrant glycosylation or oxidation), thereby generating novel antigenic epitopes.

### 2.5. Biosimilars

Unlike the biological drug categories defined based on their molecular structure or physicochemical properties—such as mAbs, ADCs, peptide drugs, glycoprotein hormones, and recombinant blood products—biosimilars are essentially a regulatory classification rather than an independent drug category based on molecular essence or pharmacophore characteristics. Given that they are a type of therapeutic biological agent, biosimilars must undergo comprehensive analytical characterization and non-clinical and clinical studies to demonstrate their high similarity in quality, safety, and efficacy to the approved reference biological drug. Their active ingredients can be mAbs, fusion proteins, or other recombinant proteins. In this section, we discuss biosimilars within the broader framework of therapeutic recombinant biological products, with a special focus on their immunogenicity characteristics compared with the reference biological drug.

#### 2.5.1. Reference Products and Biosimilars

Biosimilars are medications that highly resemble the original drugs in terms of quality, safety, and efficacy. Nevertheless, differences in manufacturing processes and quality-control strategies may result in variations in immunogenicity between the biosimilars and the originator biologics.

In eight clinical trials analyzed by Chen Xia et al. [15], ADA positivity was recorded in 46–100% of the cases in the originator adalimumab groups and 44–98.4% of the cases in the biosimilar groups. These findings suggest comparable ADA incidence rates between biosimilars and originator products. However, the immunogenicity of biosimilars must still be rigorously evaluated through comprehensive clinical trials and validated bioanalytical methods to ensure biosimilarity with respect to immune response profiles.

Moreover, although ADA positivity rates may appear comparable, substantial differences may still exist in ADA titers and NAb incidence among different products. These parameters more accurately reflect the magnitude and clinical relevance of the immune response. High ADA titers or elevated NAb positivity may compromise therapeutic efficacy by accelerating drug clearance, reducing systemic exposure, or directly neutralizing pharmacological activity. Consequently, immunogenicity assessment of biosimilars should extend beyond simple ADA incidence and include detailed characterization of antibody persistence, neutralizing capacity, and clinical impact.

#### 2.5.2. Immunogenicity Differences Among Biosimilars from Different Manufacturers

Variability in manufacturing processes and quality-control systems of biosimilar production among different manufacturers may also result in differences in the immunogenicity profiles. For example, adalimumab biosimilars developed by different companies have demonstrated substantial variation in ADA incidence during clinical evaluation [4,16]. Indeed, while some biosimilars exhibited relatively low ADA positivity rates, others showed markedly higher immunogenicity.

These differences are likely associated with variations in upstream and downstream manufacturing processes, formulation composition, purification strategies, and aggregate control. Certain manufacturers have incorporated additional purification steps capable of removing oligomeric species before aggregate growth enters the rapid propagation phase. By eliminating these oligomeric precursors early in the production stage, aggregate burden in the final product can be substantially reduced, thereby lowering the immunogenicity risk.

Such findings underscore the critical importance of robust manufacturing consistency and stringent quality control in biosimilar development. Even subtle differences in aggregation propensity, posttranslational modifications, or impurity profiles may influence immune recognition, ultimately affecting the safety and long-term efficacy of biosimilar therapeutics.

## 3. Potential Mechanisms Underlying Immunogenicity

Immunogenicity refers to the capacity of a therapeutic agent or its metabolites to elicit immune responses [17]. This phenomenon encompasses the generation of ADAs, but also multiple aspects of both innate and adaptive immunity. Innate immune responses are rapid and recognize conserved molecular patterns associated with foreign substances. As such, they mediate immediate defensive reactions through mechanisms such as the activation of PRRs, including Toll-like receptors (TLR s), and the release of inflammatory cytokines [18]. Adaptive immunity recognizes specific antigens and develops relatively slowly, but it can generate long-lasting immune memory. Adaptive responses involve both cellular and humoral immunity mediated primarily through T- and B-cell activation, ultimately leading to ADA formation [19].

Protein aggregation substantially amplifies immunogenicity by simultaneously activating innate and adaptive immune pathways. Not only does aggregation alter the structural conformation of therapeutic molecules and expose novel antigenic epitopes, but it also promotes cytokine release and enhances antigen presentation, thereby potentiating both innate and adaptive immune responses and increasing the likelihood of ADA production.

### 3.1. Innate Immunity

#### 3.1.1. Recognition of Repetitive Epitopes

The presence of repetitive epitopes within therapeutic molecules may facilitate immune recognition and activation of innate immune responses. Misfolded protein aggregates often expose antigenic epitopes distinct from those found in native proteins. These abnormal epitopes may be recognized by the immune system as foreign structures, thereby increasing the likelihood of breaking self-tolerance [20].

Aggregated proteins, including therapeutic antibodies, markedly enhance the activation of innate immune cells such as peripheral blood mononuclear cells and DCs. For example, antibody aggregates can induce upregulation of DC maturation markers—including CD80, CD86, and MHC class II molecules—while simultaneously enhancing antigen-uptake and -presentation capacity. Such changes create a highly immunostimulatory environment that favors subsequent adaptive immune activation [21].

#### 3.1.2. Cytokine Release

Protein therapeutics may aggregate during manufacturing, storage, or administration. These aggregates can be recognized by innate immune cells—including DCs and macrophages—through PRRs such as TLRs, thereby triggering inflammatory signaling pathways and promoting the release of proinflammatory cytokines, including IL-1, IL-6, and TNF-α. The resulting cytokine cascade activates innate immune responses and amplifies inflammatory signaling.

Interferon-γ (IFN-γ) produced within the local microenvironment can further activate macrophages, enhancing their phagocytic and antimicrobial functions [22]. Rituximab aggregates provide a representative example of this phenomenon. Namely, in a study comparing the effects of hGH, rituximab, and serum-purified human IgG1 aggregates on monocyte-derived DC (MoDC) activation, Gallais et al. [22] demonstrated that in vitro stimulation of MoDCs with rituximab aggregates resulted in a 2.31-fold increase in IL-12p40 secretion. As a key mediator of Th1 polarization, elevated IL-12p40 promoted IFN-γ secretion, thereby skewing adaptive immunity to-ward a cell-mediated immune phenotype and significantly increasing the risk of ADA formation.

#### 3.1.3. Recognition by PRRs

Following administration, therapeutic molecules may be recognized by cell-surface PRRs, particularly TLRs [23]. TLRs recognize pathogen-associated molecular patterns and damage-associated molecular patterns, subsequently activating MyD88-dependent NF-κB signaling pathways, which induce the release of IL-6, TNF-α, and related inflammatory mediators, thereby initiating immune activation [24].

Similarly, protein aggregates may be recognized by PRRs, leading to the activation of innate immune responses. Inhibition of TLR2 and TLR4 partially reduces cytokine secretion induced by protein aggregates, indicating that aggregated proteins likely initiate innate immune activation through PRR-mediated signaling pathways [24,25].

**Figure 3:**
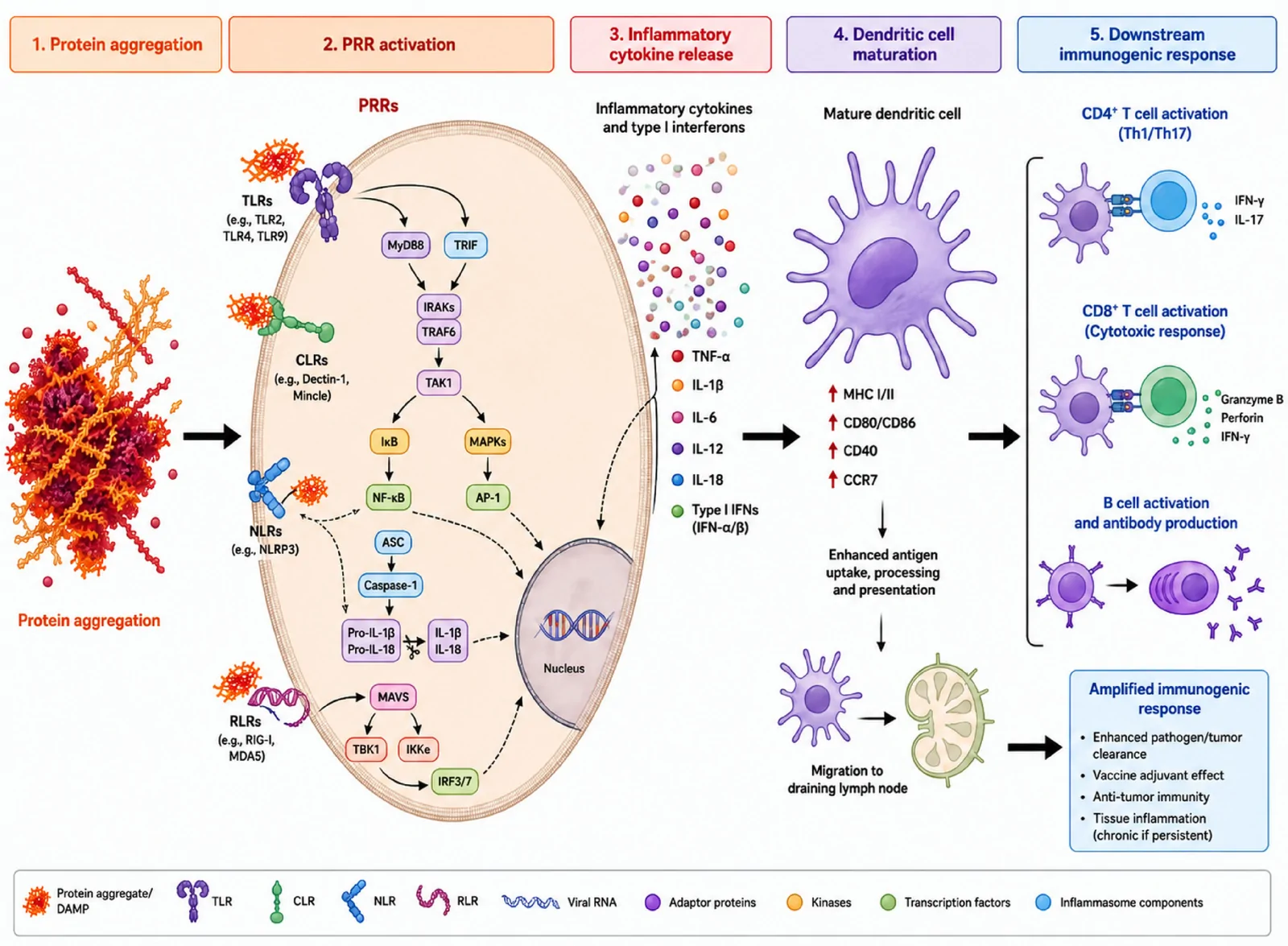
Protein aggregation is sensed by pattern-recognition receptors (PRRs) such as Toll-like receptors (TLRs), C-type lectin receptors (CLRs), and retinoic acid-inducible gene I (RIG-I)-like receptors (RLRs), which recruit the mitochondrial antiviral-signaling (MAVS) protein to assemble TBK1/IKKε for IRF3/7 phosphorylation and type-I interferon transcription, while NLRP3 inflammasome and RIP3 complexes contribute to the aggregative scaffold. Downstream PRR signaling via MyD88/TRIF sequentially activates IRAKs, TRAF6, MAPKs, NF-κB, and AP-1, coupled with ASC–Caspase 1 proteolytic activity, to drive the release of TNF-α, IL-1β, IL-6, IL-12, IL-18, and IFN-α/β. This establishes an early inflammatory milieu that promotes DC maturation and antigen presentation, ultimately activating CD4+ Th1/Th17 cells, CD8+ cytotoxic T cells, and B-cell antibody production. As a result, immunogenicity is promoted and ADA formation is initiated, though persistent inflammation may lead to chronic tissue pathology. Key nodes include NLRP3, MAVS, TBK1, and IRF3/7, which precisely outline the regulatory logic of this signaling network.

As illustrated in Figure 3, protein aggregation exerts profound effects on the innate immune system by promoting PRR activation, inflammatory cytokine release, and DC maturation, thereby initiating and amplifying downstream immunogenic responses [24].

### 3.2. Adaptive Immunity

Adaptive immunity constitutes the body’s second line of defense and is capable of recognizing and responding to specific antigens [26]. It encompasses both cellular and humoral immune responses mediated primarily by T and B cells. A defining feature of adaptive immunity is the establishment of immunological memory. Therefore, therapeutic recombinant biologics may induce long-term immune memory directed against the drug itself, thereby rendering patients more susceptible to recurrent immune responses during subsequent treatment cycles.

T and B cells recognize specific epitopes, peptides, or antigens through their respective T-cell receptors (TCRs) and B-cell receptors (BCRs). Upon antigen recognition, together with co-stimulatory signaling mediated by surface receptors and cytokines, these lymphocytes become activated and differentiate into effector cells [27].

Activation of antigen-specific CD4+ helper T (Th) cells promotes the activation and proliferation of cognate antigen-specific B cells, which subsequently differentiate into antibody-producing plasma cells. This process is referred to as T-cell-dependent antibody production. Antibody generation may also occur through T-cell–independent pathways.

#### 3.2.1. Antigen Presentation

Therapeutic molecules may be internalized by APCs and subsequently presented to T cells, thereby initiating adaptive immune responses [28]. T cells recognize antigenic peptides presented within MHC molecules through their surface TCRs. T-cell activation occurs only when the TCR specifically engages the peptide–MHC complex [29].

DCs are professional APCs capable of highly efficient antigen uptake, processing, and presentation, thereby activating naïve T cells [30]. Protein aggregation can enhance uptake and presentation of therapeutic molecules by APCs, resulting in amplified adaptive immune activation. Aggregation has also been shown to markedly promote DC maturation.

Certain recombinant protein therapeutics may additionally trigger autoimmune reactions through aberrant T-cell activation directed against self-tissues. Aggregated proteins—including monoclonal antibodies and recombinant proteins—have been shown to significantly increase expression of DC surface markers such as CD80, CD86, and MHC class II molecules. Aggregated hGH, for instance, induced substantially higher expression of CD80, CD86, and MHC II on MoDCs compared with monomeric hGH [22].

Protein aggregation also markedly enhances APC function. Aggregated proteins are more readily internalized and presented to T cells than their native counterparts. In vitro studies using rituximab as a model antibody demonstrated that following uptake of aggregated rituximab by MoDCs, MHC II expression and antigen presentation capacity were significantly enhanced [31].This effect may arise because aggregated proteins possess larger surface areas and more structurally complex conformations, facilitating receptor recognition and binding by APCs.

#### 3.2.2. Fc Receptor-Related Responses

Fc receptors (FcRs) play critical roles in immune regulation, and aggregated recombinant proteins may activate adaptive immune responses through interactions with FcRs. Zhao Ruijie et al. stimulated mouse peritoneal macrophages with an anti-FcγRIIb crosslinking antibody and observed upregulated mRNA expression of multiple proinflammatory mediators, including TLR3, IRF3, IFN-α, IFN-β, and IFN-γ [32]. Notably, this finding appears to contradict the conventional understanding of FcγRIIb as an inhibitory receptor transducing signals via its ITIM motif. This discrepancy may be attributable to nonspecific co-crosslinking of murine activating Fcγ receptors by the Fc portion of the crosslinking antibody used in the experiment, or it may reflect non-canonical signaling of this receptor under specific cellular microenvironments.

In addition, binding of aggregated recombinant proteins to FcRs may promote antibody-dependent cellular cytotoxicity (ADCC) and antibody-dependent cellular phagocytosis (ADCP), thereby further intensifying immune activation [33,34]. Consequently, FcR-mediated pathways represent important contributors to aggregation-induced immunogenicity and should be carefully considered during therapeutic development.

#### 3.2.3. Generation of ADAs

Protein aggregation is one of the principal factors contributing to ADA formation. Aggregated recombinant proteins may expose novel antigenic epitopes capable of activating B cells and inducing ADA production [35]. In addition, aggregated proteins can stimulate generation of IgG2a antibodies, indicating activation of T-cell-dependent immune responses.

ADA formation not only diminishes therapeutic efficacy but may also provoke severe immune-mediated adverse events, including hypersensitivity reactions and CRS. Therefore, controlling protein aggregation is essential for minimizing ADA generation.

The term ADA broadly refers to all antibodies capable of binding therapeutic drug molecules, irrespective of whether they alter drug function. The proportion of ADAs with neutralizing activity varies by therapeutic: for anti-TNF-α agents such as adalimumab, golimumab, and certolizumab, >97% of ADAs target the TNF binding site and function as neutralizing antibodies, whereas for infliximab, approximately >90% of ADAs exhibit neutralizing activity [36]. Neutralizing ADAs directly attenuate or abolish therapeutic efficacy by blocking target binding, interfering with molecular conformation, or accelerating drug clearance, while non-neutralizing ADAs may still alter pharmacokinetics through immune-complex formation [37]. Although non-neutralizing ADAs do not directly interfere with pharmacological activity, they may still alter pharmacokinetics through immune-complex formation, accelerating drug clearance, reducing bioavailability, and lowering effective drug concentrations, thereby ultimately contributing to therapeutic failure.

## 4. Influence of Drug-Related Factors on Aggregation and Immunogenicity

Therapeutics at different stages of their shelf life exhibit distinct immunogenic characteristics. Newly manufactured products generally display relatively low immunogenicity owing to stringent manufacturing controls and comprehensive clinical evaluation. During the approved shelf life period, the composition and structural integrity of the drug remain comparatively stable; however, fluctuations in storage conditions may induce mild degradation or denaturation, thereby influencing immunogenicity [37]. As products approach the end of their shelf life, prolonged storage may lead to degradation and aggregation, substantially increasing the immunogenic risk.

### 4.1. Comparative Immunogenicity Across Different Shelf Life Stages

#### 4.1.1. Newly Manufactured Products

The clinical evaluation of newly manufactured therapeutics reflects the high priority placed on immunogenicity assessment. Clinical trials are typically designed in multiple stages and layers to comprehensively evaluate safety and efficacy in humans [38]. In Phase I studies, investigators primarily assess tolerability and preliminary pharmacokinetic characteristics while simultaneously monitoring immunological parameters—such as antibody formation rates and the magnitude of cellular immune responses—to evaluate potential immunogenicity risks [39].

Upon progression to Phase II and III trials, expanded study populations and larger sample sizes enable further validation of immunogenicity across broader patient cohorts. Selection of immunogenicity end points is also critical. Commonly employed indicators include ADA positivity rates, NAb levels, and immune-complex formation, all of which collectively reflect the extent of immune stimulation induced by the therapeutic agent [40]. Systematic clinical evaluation can identify the potential risk factors of immunogenicity, thereby providing a scientific basis for the optimization of formulations and manufacturing processes and ensuring the low-immunogenicity profile of newly manufactured products.

#### 4.1.2. Within the Approved Shelf Life Period

Under recommended storage conditions, the immunogenicity of pharmaceuticals generally remains relatively stable throughout the approved shelf life period. However, fluctuations in storage conditions or exposure to extreme environments may introduce mild changes. These variations are governed by the dynamic interplay among intrinsic molecular stability, storage conditions, and duration of storage.

From the perspective of intrinsic drug stability, therapeutics with chemically stable structures and rational formulation designs typically exhibit minimal changes in immunogenicity during storage, whereas structurally complex or less stable products are more susceptible to immunogenic fluctuations. External environmental factors—including temperature, humidity, and light exposure—also exert nonlinear effects on immunogenicity [41]. Within optimal temperature ranges, immunogenic changes tend to occur slowly; however, once storage conditions exceed acceptable thresholds, the rate of immuno-genic alteration may increase substantially. Long-term fluctuations in storage conditions may additionally lead to cumulative molecular degradation, exerting clinically meaningful effects on immunogenicity.

It has been shown that even under standard storage conditions, certain therapeutics exhibit slight increases in immunogenicity as the end of their shelf life approaches, potentially due to accumulation of degradation products and protein aggregates over prolonged storage [21]. Different storage conditions can yield aggregates with distinct immunogenic profiles, and aggregates formed during long-term storage may exhibit enhanced immunogenicity compared with those in freshly manufactured products [36]. Overall, the immunogenicity profile of drugs during the shelf life period reflects a dynamic equilibrium between molecular stability and environmental exposure. Under ideal storage conditions, immunogenicity remains low and relatively stable, whereas in non-ideal conditions, the immunogenic risk is significantly elevated. Consequently, to minimize immunogenic alterations and ensure therapeutic safety and efficacy, it is essential to optimize storage environments and reinforce storage management.

#### 4.1.3. Near Expiration Date

The near expiration stage refers to the period approaching the labeled expiration date of a pharmaceutical product. Prolonged storage increases the likelihood of degradation and aggregation during this stage, thereby elevating immunogenicity. For example, degradation of peptide therapeutics may generate reactive intermediates containing highly reactive functional groups that are capable of covalently binding proteins or other biomacromolecules, ultimately resulting in highly immunogenic complexes.

The increased immunogenic risk associated with near expiration therapeutics primarily arises from chemical and physical alterations occurring during long-term storage. From chemistry perspective, degradation commonly involves hydrolysis, oxidation, and isomerization reactions, which are closely linked to molecular structural characteristics. Drugs containing ester or amide bonds are particularly susceptible to hydrolysis under high-humidity conditions, whereas compounds containing phenolic hydroxyl groups or polyunsaturated bonds are more prone to oxidative degradation. Temperature also exerts profound effects on degradation kinetics. According to the Arrhenius equation, elevated temperatures exponentially increase reaction rate constants, thereby accelerating degradation processes.

From a physical standpoint, aggregation stems primarily from enhanced intermolecular interactions and solution-phase separation phenomena. During storage, solvent evaporation, pH variation, or changes in ionic strength may promote protein self-association and the formation of large aggregates. Not only does such aggregation alter the physical state of the drug, but it may also modify surface characteristics, thereby further influencing immunogenicity.

### 4.2. Comparative Immunogenicity Across Different Routes of Administration

#### 4.2.1. IV Administration

IV injection is one of the principal routes for administering therapeutic recombinant biologics and is generally considered to possess relatively low immunogenicity. Because the drug enters systemic circulation directly, it is rapidly diluted by the blood-stream, reducing local tissue concentrations and minimizing direct interaction with immune cells. This dilution effect decreases the likelihood of immune activation. Furthermore, IV administration enables rapid attainment of therapeutic steady-state concentrations, resulting in more uniform distribution and reducing the risk of immune responses triggered by localized high drug concentrations.

Nevertheless, IV administration is not entirely devoid of immunogenic risk, which is additionally influenced by drug-specific characteristics, patient immune status, and dosage [42]. In clinical practice, IV therapeutics are frequently mixed with other agents or infusion vehicles. Incompatible admixtures may alter the physical or chemical properties of the drug and induce or potentiate protein aggregation.

It has been demonstrated that dilution of certain IgG4 mAbs in different IV infusion bags reduces their physical stability, leading to the formation of visible or subvisible particles [43]. Protein aggregates may be recognized by the immune system as “nonself,” thereby inducing ADA formation [21]. These antibodies reduce circulating drug concentrations, but they may also provoke infusion-related reactions, including CRS and hypersensitivity responses. Repeated IV administration of aggregated therapeutics may sustain ADA production, progressively reducing therapeutic efficacy while increasing the risk of adverse events.

Clinically, some patients treated with IFN-β experience diminished therapeutic responses accompanied by increased disease activity. Subsequent investigations have revealed the presence of NAbs associated with protein aggregation occurring during storage or administration [21]. Aggregated IFN-β enhances immunogenicity and promotes ADA formation, thereby accelerating drug clearance while simultaneously blocking receptor binding and downstream signaling, ultimately resulting in irreversible loss of efficacy.

#### 4.2.2. SC Administration

SC formulations often possess high protein concentrations. Under such conditions, proteins may aggregate locally owing to physicochemical properties or tissue microenvironmental factors, thereby increasing immunogenicity. In addition, SC-administered drugs are absorbed more slowly and remain in local tissues for prolonged periods, which increases opportunities for their interaction with immune cells [44].

Following SC injection, therapeutics may initially drain through lymphatic vessels before being captured by skin-resident DCs or Langerhans cells. Upon maturation, these APCs migrate to draining lymph nodes, where they present antigens to T cells and initiate adaptive immune responses. The lymphatic drainage mechanism may further enhance immune activation and ADA formation.

Although no statistically significant difference in ADA incidence has been shown between IV and SC administration (*p* = 0.81; from a pooled analysis of 17 proteins; initial 7-protein dataset *p* = 0.55), mechanistic studies suggest that SC administration may increase the immunogenic risk because of high local drug concentrations, slow absorption kinetics, and lymphatic trafficking [45]. In particular, local aggregation within tissues may intensify immune activation and promote ADA generation. Indeed, several therapeutic proteins exhibit higher ADA incidence following SC administration, which may be attributed to local aggregate formation.

#### 4.2.3. Intravitreal Administration

Numerous antibody-based therapeutics and recombinant proteins—including the humanized Fab fragment ranibizumab, the full-length anti-VEGF antibody bevacizumab, and aflibercept, a VEGFR1/2 extracellular domain-IgG1 Fc fusion protein—are widely used to treat ocular disorders such as diabetic retinopathy [46]. Because the eye possesses immune-privileged properties that suppress excessive immune activation and prevent tissue damage, intravitreal administration is generally associated with relatively low immunogenicity.

However, the high local drug concentration, specialized ocular immune environment, small tissue volume, and extreme tissue sensitivity may produce unique immune responses [47]. Although intravitreally administered drugs primarily act locally, they may diffuse through intraocular circulation into retinal and choroidal vessels, thereby contacting immune components. Drug diffusion into retinal or choroidal tissues is partially restricted by tight junctions of retinal pigment epithelial cells.

Despite ocular immune privilege, intravitreal therapeutics may still provoke local immune reactions, including noninfectious inflammation, retinal injury, or intraocular hemorrhage. Recombinant protein therapeutics themselves may function as foreign antigens and could be internalized by residual APCs within the vitreous or subretinal space, including microglia, DCs, and retinal pigment epithelial cells. These antigens may subsequently be presented via MHC class II pathways to infiltrating T cells, initiating adaptive immunity.

Temperature fluctuations, silicone oil–protein complexes, or protein aggregation during handling and injection may substantially increase immunogenicity and trigger sterile vitritis. Drug aggregates or viral vector-derived nucleic acids may additionally activate PRRs, such as TLRs and RIG-I, thereby stimulating NF-κB and IRF signaling pathways and inducing inflammatory cytokines such as IL-1β, TNF-α, and IFN-γ. Formation of ADAs may lead to immune-complex deposition within retinal or choroidal vessels, triggering type-III hypersensitivity reactions characterized by retinal vasculitis, choroidal thickening, or vascular leakage. Activated CD4+ Th1/Th17 cells release IFN-γ and IL-17, further disrupting tight-junction proteins and exacerbating blood–retinal barrier damage, ultimately creating a vicious cycle of inflammation, vascular leakage, increased drug exposure, and intensified immune responses. Clinically, repeated injections are often associated with progressively earlier and more severe inflammatory episodes.

#### 4.2.4. Pulmonary Administration

Compared with SC delivery, pulmonary administration represents an emerging route for protein therapeutics. Although varying levels of protein aggregates may not significantly alter immunogenicity individually, the overall immunogenicity associated with pulmonary delivery remains relatively high [44]. This may reflect the heightened sensitivity of pulmonary immune surveillance and the unique deposition and distribution patterns of proteins within the lungs.

When protein therapeutics deposit on alveolar surfaces, they initially encounter airway surface fluid. If homeostatic balance is disrupted—for example, by protein aggregation—immune cells become activated and release large quantities of proinflammatory cytokines, including IL-1β, IL-6, TNF-α, and type-I interferons [43]. Once initiated, this inflammatory cascade can disrupt baseline immune tolerance.

Mahri et al. [48] assessed the immunogenicity of spray-dried NIP228 formulations with varying aggregate levels following intratracheal administration in mice. While pulmonary administration evoked a more robust immune response than SC administration, the magnitude of the ADA response was not significantly affected by the differences in aggregate levels, suggesting that protein aggregation was not the primary driver of immunogenicity following pulmonary delivery. Similarly, Ratanji et al. [35] reported that intraperitoneal injection of aggregated scFv fragments or ovalbumin in mice induced significantly higher IgG2a antibody levels than injection of their monomeric forms.

When proteins form nonnative aggregates or are accompanied by danger-associated signals—such as reactive oxygen species generation or NLRP3 inflammasome activation—immune tolerance may be disrupted, promoting Th2- or Th17-skewed responses and increasing the risks of hypersensitivity and ADA formation. Th2-dominant responses are associated with allergic manifestations, including asthma-like reactions, whereas Th17-driven responses promote intense autoimmune inflammation and markedly increase the likelihood of ADA development [22].

## 5. Immunogenicity Induced Under Different Stress Conditions

### 5.1. Thermal Stress

Temperature is one of the most critical factors influencing the immunogenicity of therapeutic recombinant biologics. Exposure to elevated or reduced temperatures may induce protein degradation or aggregation, thereby increasing immunogenicity. Elevated temperatures initially disrupt intramolecular hydrogen bonds; when the temperature approaches or exceeds the protein melting temperature (Tm), α-helical structures may undergo pronounced unwinding, and certain regions may transition from α-helices to β-sheet conformations, thereby creating hydrophobic interfaces that promote subsequent aggregation. Simultaneously, expansion and destabilization of hydrophobic cores expose aromatic and hydrophobic side chains, detectable as a red shift in intrinsic tryptophan fluorescence.

Therefore, high temperatures may expose normally buried hydrophobic regions, thereby triggering aggregation. Although low-temperature storage can slow degradation kinetics, freeze–thaw cycles may reduce solvent polarity and expose hydrophobic amino-acid residues, thereby inducing conformational changes that also promote aggregation [49]. Taken together, thermal stress significantly compromises the stability of recombinant proteins and related biologics by progressively disrupting higher-order structures, ultimately leading to loss of activity, aggregation, or precipitation.

### 5.2. Mechanical Stress

Mechanical stress is a common external factor encountered during pharmaceutical manufacturing and storage. Such stress can markedly affect protein stability and alter immunogenicity. When protein solutions are exposed to air–liquid or liquid–solid interfaces, hydrophobic cores tend to migrate toward and adsorb onto low-energy hydrophobic interfaces, resulting in local conformational unfolding rather than the global unfolding typically induced by heat stress [50].

Unlike the relatively disordered aggregates produced by thermal stress, mechanically induced interfacial aggregates possess a dual immunostimulatory mechanism involving both “physical morphology” and “conformational signaling” [24]. Not only do these aggregates provide multivalent antigen presentation that promotes B-cell activation, but they also expose hydrophobic epitopes capable of activating innate immune pathways as danger signals. Owing to this dual signaling capacity, mechanically stressed samples containing relatively few subvisible particles can still induce robust ADA responses, typically skewed toward a Th1-dominant immune profile characterized by stronger cellular immunity, in contrast to the predominantly humoral immune responses associated with thermal stress [35].

### 5.3. Chemical Stress

Chemical stressors—including pH fluctuations and oxidative agents—also profoundly affect protein stability and immunogenicity. When the pH approaches the protein isoelectric point (pI), the net surface charge becomes approximately neutral, reducing in-termolecular electrostatic repulsion and facilitating protein–protein interactions that promote aggregation and formation of disordered aggregates.

Oxidative stress can further promote protein oxidation, which leads to the formation of dimers or higher-order oligomers that increase immunogenicity. Metal ions such as Cu^2+^ and Fe^3+^, as well as reactive oxygen species, can directly oxidize methionine, tryptophan, and related residues, generating carbonyl derivatives and incorrect disulfide-bond pairing, ultimately producing fragment–aggregate complexes [51]. For example, rh-insulin readily aggregates when disulfide-bond formation is impaired or misplaced—such as under excessive oxidative conditions, insufficient reducing environments, or in ammonium bicarbonate buffer systems—resulting in highly immunogenic particulate species.

Chemical modifications, including glycosylation and PEGylation, can also alter physicochemical properties, thereby affecting protein stability and immunogenicity. Glycosylation, in particular, enhances both colloidal and conformational stability and reduces aggregation, representing a major mechanism by which immunogenicity is mitigated. Glycan chains attached to potential B-cell epitopes can physically shield these regions, thereby preventing antibody- or immune-receptor recognition and reducing the likelihood that the therapeutic protein will be identified as a foreign antigen.

### 5.4. Light Stress

Light stress, particularly ultraviolet (UV) irradiation, exerts significant effects on the stability and immunogenicity of therapeutic recombinant proteins. Upon absorption of UV photons, electrons transition from the ground state to an excited state [52]. Molecules in these high-energy excited states become unstable, with intensified bond vibrations that promote free-radical formation and cleavage of chemical bonds within protein molecules. Such light-induced chemical reactions may generate novel antigenic epitopes capable of activating the immune system and increasing immunogenicity.

It has been shown in vivo that UV-exposed recombinant proteins are more readily recognized by immune cells, leading to enhanced immune activation and significantly increased ADA formation [1]. Accordingly, to reduce the immunogenicity risk, excessive UV exposure should be minimized throughout the manufacturing, storage, and clinical handling of therapeutic recombinant proteins.

## 6. Influence of Patient-Specific Factors on Immunogenicity

### 6.1. Physiological Factors

The overall “activity level” of the immune system largely determines the magnitude of immunogenic responses. Therefore, baseline immune status is a critical determinant of immunogenicity [53]. Patients with autoimmune diseases—such as rheumatoid arthritis or systemic lupus erythematosus—typically possess highly activated immune systems and are more prone to developing ADAs [54]. This heightened susceptibility is closely associated with elevated inflammatory cytokine levels and enhanced APC activity.

In contrast, immunosuppressed patients—such as those on chemotherapy or immunosuppressive therapies—may exhibit a reduced immunogenicity risk. For example, glucocorticoids and azathioprine suppress DC maturation and T-cell activation. Their combined use has been clinically linked to a significant reduction in ADA positivity rates. In infliximab therapy, addition of immunomodulatory agents represents an effective strategy for mitigating immunogenicity. Clinical studies have shown that patients receiving infliximab in combination with azathioprine exhibit an ADA incidence of only 0.9%, compared with 14.6% in patients treated with infliximab monotherapy [55].

### 6.2. Genetic Factors

The HLA system plays a central role in antigen presentation, and HLA polymorphisms have been associated with an individual’s capacity for immune recognition of therapeutic agents. However, immunogenicity is a multifactorial phenomenon. Indeed, while genetic factors—including those encoding proteins involved in immune regulation and HLA class II–mediated antigen presentation—appear to substantially affect the immunogenicity risk, non-genetic factors such as formulation, dosing regimen, and concomitant medications also play important roles and may interact with genetic susceptibility.

Certain HLA molecules display higher affinity for specific epitopes and more readily activate T-cell-dependent immune responses, although the causal relationship between specific HLA alleles and immunogenicity remains incompletely understood. The highest risk has been observed in patients carrying HLA-DQA1*05 who receive infliximab monotherapy, in whom the cumulative 1-year incidence of ADAs may reach 92% [56]. However, these findings should be interpreted with caution, given that HLA-linked immunogenicity is likely multifactorial and may be influenced by additional genetic polymorphisms, environmental triggers, and methodological limitations inherent to association studies.

In addition, interindividual variation in the T-cell repertoire influences the magnitude of immune responses [54]. Differences in prior infections, vaccination history, and gut microbiota composition may alter T-cell recognition of therapeutic epitopes, potentially leading either to the loss of immune tolerance or excessive immune activation.

### 6.3. Combination Therapy

Co-administration of immunosuppressive agents can effectively reduce ADA formation. There is growing evidence that the abundance and biological characteristics of drug targets significantly influence the immunogenicity of mAbs and other therapeutic proteins [57]. When target abundance is low, a larger proportion of free therapeutic antibody remains unbound and is, therefore, more readily internalized and presented by APCs, increasing the likelihood of immune activation. In contrast, highly abundant targets can effectively “absorb” greater quantities of therapeutic antibodies, reduce exposure of free antibodies, and thereby lower the immunogenicity risk.

The nature of the target also plays an important role. Antibodies against membrane-bound antigens, such as CD20 or CD52, form mAb–antigen complexes that are more efficiently internalized by APCs, thereby eliciting stronger immune responses. In contrast, antibodies targeting soluble proteins, including TNF-α or IL-6, generally form smaller immune complexes, which are cleared more rapidly and are therefore associated with lower immunogenicity [58]. If targets are highly expressed within lymphoid tissues or APC-rich regions—such as the spleen or lymph nodes—local immune activation is more readily induced. Certain targets, including CD52, are expressed directly on APC surfaces; binding of therapeutic antibodies to these targets may directly activate APCs, thereby substantially increasing the immunogenicity risk.

Combination therapy with nivolumab (targeting PD-1) and ipilimumab (targeting CTLA-4) produces higher ADA incidence rates than nivolumab monotherapy. In a model-informed immunogenicity assessment pooling data from 28 clinical trials (*n* = 7820), nivolumab’s ADA incidence was approximately 16% for monotherapy and 32% for combination therapy [59].

Collectively, lower target abundance, stronger membrane localization, and increased likelihood of APC interaction are associated with enhanced immunogenicity. Therefore, target-related characteristics should be regarded as a core dimension in immunogenicity risk assessment during both therapeutic design and clinical strategy development.

## 7. Strategies for Reducing the Occurrence of Aggregates and Lowering the Risk of Immunogenicity

### 7.1. Optimization of Manufacturing Processes

#### 7.1.1. Control of Manufacturing Environmental Factors

##### Temperature Control

During the manufacturing of protein therapeutics, temperature plays a pivotal role in maintaining protein stability. When temperatures approach the protein melting temperature or cold denaturation threshold, thermodynamic stability decreases substantially and structural fragility reaches a critical point. In such conditions, even minor environmental fluctuations may trigger unfolding and aggregation. These conformational alterations increase the likelihood that the immune system will recognize the protein as “nonself.”

Elevated temperatures may induce protein denaturation and aggregation, whereas low temperatures, although capable of slowing down degradation, may also promote aggregation if improperly managed. During lyophilization, rapid freezing can reduce cold denaturation; however, it also generates small ice crystals, thereby increasing the total ice–water interfacial area. For proteins prone to surface-induced denaturation, such conditions may be suboptimal. Consequently, lyophilization parameters—including freezing rate and the incorporation of annealing steps—must be optimized individually for each protein formulation so as to minimize the aggregation risk and preserve structural integrity.

##### pH Control

Changes in pH profoundly influence protein conformational stability. Protein secondary structures, including α-helices and β-sheets, as well as overall folding states, may shift in response to pH variation [60]. Accordingly, precise control of formulation pH and ionic conditions during manufacturing is essential to maintain protein solubility and conformational stability while minimizing propensity to aggregation.

##### Ionic Strength Control

Ionic strength regulates electrostatic interactions between protein molecules primarily by altering surface charge distribution. Not only does increased ionic strength affect static protein structure, but it also profoundly influences aggregation kinetics. Elevated ionic strength weakens electrostatic repulsion, enabling protein molecules to approach each other more readily and thereby accelerating aggregation processes [61].

Furthermore, ionic strength can alter aggregation pathways and influence the morphology of the resulting aggregates. By modulating electrostatic interactions, secondary structure stability, and aggregation kinetics, high ionic strength promotes protein aggregation and may adversely affect biological function. Therefore, careful regulation of ionic strength is essential in both biomedical and industrial applications to preserve protein stability and functionality.

#### 7.1.2. Chemical Modification

##### PEGylation

PEGylation, the covalent conjugation of polyethylene glycol (PEG) to proteins, is a widely used modification strategy for improving protein therapeutics. PEGylation can extend plasma half-life, thereby enhancing therapeutic efficacy. However, the effects of PEGylation vary depending on the specific protein involved.

Importantly, PEGylation may also alter protein conformational structure and expose novel epitopes to the immune system, potentially eliciting immune responses. Thus, although PEGylation can improve protein stability and pharmacokinetic performance, its potential effects on immunogenicity must be carefully balanced during formulation design.

##### Glycosylation and Glycation

Glycosylation is one of the most common posttranslational modifications of proteins and exerts profound effects on protein stability, function, and immunogenicity. Aberrant glycosylation patterns may introduce novel antigenic epitopes, thereby increasing the immunogenicity risk. In contrast, appropriate glycosylation generally suppresses aggregation by enhancing both colloidal and conformational stability, thereby reducing immunogenicity at the physicochemical level.

Human serum albumin, one of the most abundant plasma proteins, readily undergoes glycation, which can alter both its structure and biological function. Unlike enzymatic N-glycosylation of IgG, albumin undergoes nonenzymatic glycation (e.g., by glucose or methylglyoxal), which can promote aggregation. Similarly, glycated bovine serum albumin exhibits increased aggregation propensity [62,63].

**Figure 4:**
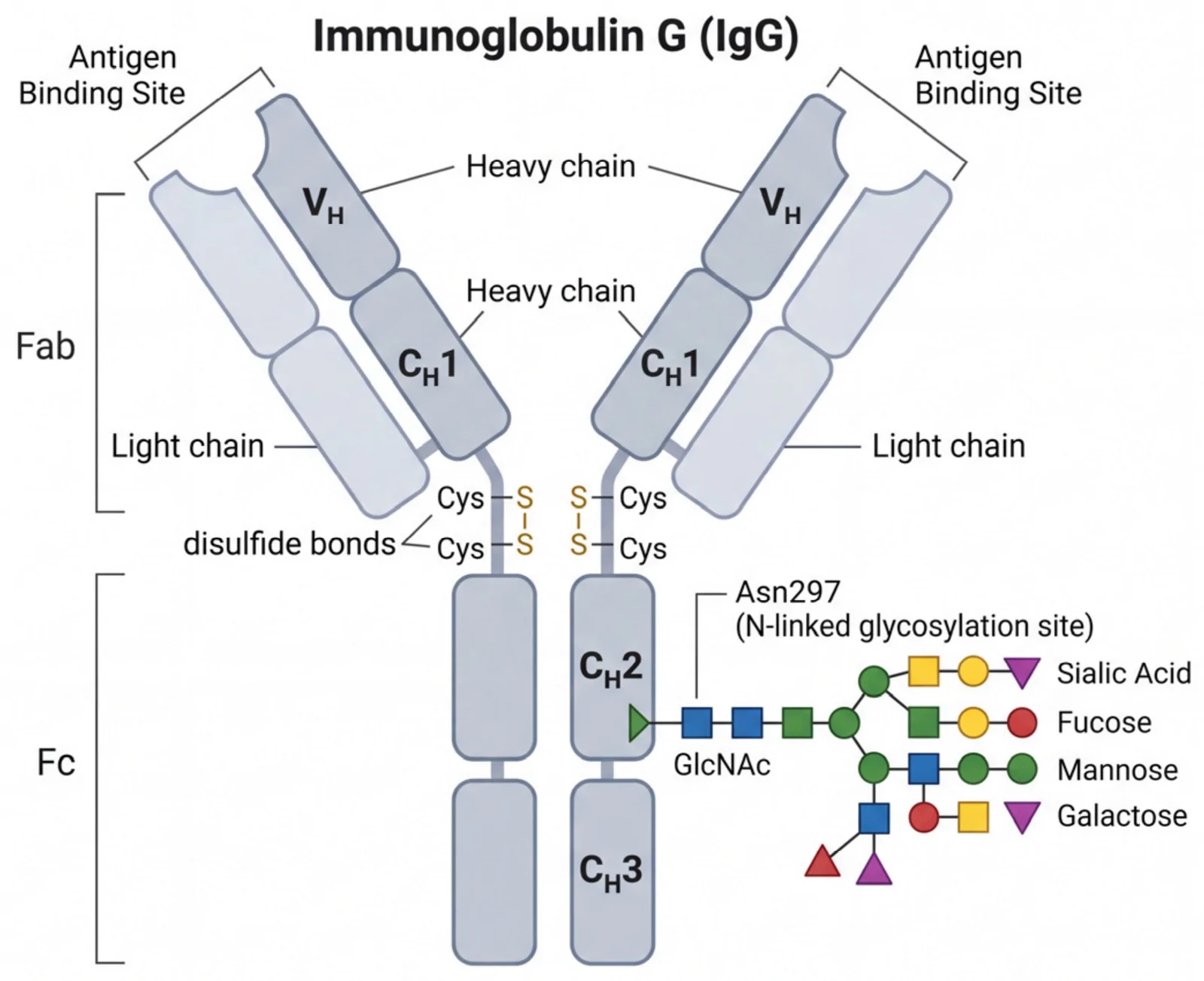
Diagram of immunoglobulin (IgG) antibody structure, highlighting the highly conserved N-linked glycosylation site Asn297 in the CH2 domain of the Fc region and its glycan composition (including GlcNAc, mannose, galactose, fucose, and sialic acid) [21]. The IgG antibody consists of two light chains and two heavy chains, each with V and C domains. The Fab is responsible for antigen recognition, while the Fc mediates effector functions. These two fragments are connected by a hinge region containing disulfide bonds. Furthermore, the diagram indicates common conjugation strategies for antibody–drug conjugate (ADC) design: site-specific conjugation via reduction of hinge disulfide bonds to expose free thiols, and random conjugation by reacting active ester-modified drugs with lysine residues widely distributed across the domains. This figure was constructed using Servier Medical Art resources to clarify the structural features of IgG and key molecular targets for ADC development. In the glycan legend, blue squares represent GlcNAc, green circles indicate mannose, red circles indicate galactose, yellow circles indicate fucose, and purple inverted triangles indicate sialic acid.

For IgG antibodies, a highly conserved N-glycosylation site is located within the CH2 domain of the Fc region, as illustrated in Figure 4. Glycosylation at this site is associated with enhanced Fc receptor interactions, protection against proteolytic degradation, and reduced aggregation tendency. The impact of glycosylation on protein stability and aggregation is highly dependent on glycan composition and chain size. Therefore, during recombinant antibody production, glycosylation processes must be precisely regulated and glycan structures should be optimized to ensure antibody stability and biological activity while minimizing aggregation and functional loss.

### 7.2. Incorporation of Excipients

The incorporation of stabilizing excipients, such as sucrose and mannitol, represents an effective strategy for reducing intermolecular aggregation in therapeutic protein formulations. In mAb preparations, the inclusion of appropriate stabilizers can substantially enhance physicochemical stability and suppress aggregate formation, thereby mitigating the immunogenicity risk.

#### 7.2.1. Saccharide Excipients

Saccharides are among the most widely utilized stabilizers in protein formulations. Sucrose and trehalose, two representative disaccharides, primarily exert their stabilizing effects through the preferential exclusion mechanism, which enhances protein conformational stability, minimizes protein–protein interactions, and consequently reduces aggregation propensity. In liquid protein formulations, trehalose demonstrates a more pronounced preferential exclusion effect than sucrose. However, in frozen solutions, trehalose exhibits a greater tendency toward phase separation and crystallization (forming dihydrate crystals more readily than sucrose) [64], potentially compromising its stabilizing capacity. This behavior is specific to the frozen state and contrasts with lyophilized glassy trehalose, which exhibits greater resistance to crystallization.

Accordingly, the selection of saccharide excipients should be tailored to the specific formulation context, such as liquid or lyophilized preparations, so as to minimize the adverse effects of aggregation on immunogenicity. Enhanced conformational stability decreases the likelihood of protein unfolding under environmental stress, thereby reducing aggregate formation. From an immunogenicity perspective, suppression of aggregation diminishes the exposure of potential antigenic epitopes to the immune system, ultimately lowering the probability of immune activation.

Previous studies have further demonstrated that histidine-buffered formulations effectively reduce aggregation and degradation of mAbs during storage [65]. Such formulation optimization may contribute to improved product quality and stability, although its direct impact on pharmacokinetic half-life and immunogenicity requires further investigation.

#### 7.2.2. Preservatives (Benzyl Alcohol)

Benzyl alcohol is widely employed as an antimicrobial preservative in protein formulations due to its ability to effectively inhibit microbial contamination. Nevertheless, there is growing evidence suggesting that benzyl alcohol may promote partial unfolding and aggregation of certain proteins. Proteins in an aggregated state are more readily recognized by the immune system as foreign entities, thereby triggering immune responses and inducing the generation of ADAs, which may ultimately compromise therapeutic efficacy.

Consequently, preservative selection in protein drug formulation should be approached with caution. Formulation strategies should seek to minimize the use of preservatives that may facilitate protein aggregation or, alternatively, explore substitute antimicrobial approaches capable of maintaining sterility without adversely affecting protein stability.

#### 7.2.3. Surfactants

Nonionic surfactants, including polysorbate 20 and polysorbate 80, are commonly incorporated into protein formulations to prevent aggregation induced by mechanical agitation and freeze–thaw stress [66,67]. These surfactants stabilize proteins primarily by competitively adsorbing onto hydrophobic interfaces—such as container surfaces, ice–water interfaces, and air–liquid interfaces generated during agitation—thereby shielding proteins from interfacial denaturation.

The stabilizing effect of polysorbates stems from their amphiphilic molecular architecture, which enables preferential accumulation at phase boundaries. By occupying interfacial sites, polysorbates lower interfacial tension and reduce the thermodynamic driving force for protein adsorption and unfolding. [68].

The clinical significance of surfactant selection is underscored by the Eprex^®^ case study, in which the replacement of human serum albumin with polysorbate 80 and glycine was linked to increased protein aggregation and the emergence of neutralizing anti-EPO antibodies, leading to pure red-cell aplasia [69]. This landmark event illustrates that while surfactants can provide essential interfacial protection, their selection requires careful evaluation of both stabilizing and potential destabilizing effects, including interactions with container components and susceptibility to degradation.

Notwithstanding their beneficial effects, commercially available polysorbate preparations are susceptible to spontaneous auto-oxidation, leading to the formation of reactive peroxide species. These oxidative byproducts may damage protein molecules and compromise structural stability, subsequently promoting aggregation through mechanisms distinct from interfacial stress. Therefore, the purity, oxidative stability, and degradation profile of surfactants must be carefully controlled to ensure their protective function while minimizing the risk of aggregation and immunogenicity arising from surfactant-derived degradation products. Therefore, formulation development should include rigorous assessment of surfactant quality attributes and their impact on product stability throughout the product lifecycle.

### 7.3. Control of Storage and Transportation Conditions

During storage and transportation, therapeutic recombinant biologics are highly susceptible to environmental stressors, such as fluctuations in temperature and humidity, mechanical stress, and pH variation, all of which may promote protein aggregation. Once aggregation occurs, aggregated proteins often exhibit reduced conformational stability and become increasingly vulnerable to further aggregation or degradation under adverse conditions, thereby amplifying the immunogenicity risk.

For example, elevated temperatures may induce conformational unfolding, leading not only to aggregation but also to chemical degradation reactions such as oxidation and deamidation, which can subsequently provoke immune responses and compromise both drug efficacy and safety. Similarly, extreme pH conditions may disrupt intramolecular interactions, resulting in protein denaturation or aggregation and thereby increasing the immunogenic potential. Mechanical stresses generated during storage and transportation, including shaking and vibration, produce shear forces capable of destabilizing protein structure and promoting aggregate formation, ultimately enhancing the probability of immune activation.

Hence, stringent environmental control throughout storage and distribution is essential to minimize aggregation, reduce the immunogenicity risk, and ensure the safety, stability, and therapeutic efficacy of recombinant biologic products.

### 7.4. Monitoring of Aggregation Levels

Protein aggregation is frequently accompanied by alterations in higher-order structure. Therefore, continuous monitoring of aggregation status is critical for the early detection and management of aggregation-related instability. Advanced analytical technologies have become indispensable tools for characterizing aggregation behavior. Table 2 provides a comprehensive comparison of these and other analytical methods for subvisible and visible particle detection, including their measurement principles, applicable size ranges, strengths, and limitations.

In addition, spectroscopic approaches—including circular dichroism spectroscopy, particle-size analysis, and infrared spectroscopy—can be employed to monitor alterations in protein secondary structure, tertiary structure, and hydrodynamic dimensions in real time, thereby enabling early identification of aggregation events. Nuclear magnetic resonance spectroscopy and mass spectrometry have similarly been extensively applied for the structural characterization of aggregated proteins. Collectively, these analytical approaches allow detection of aggregation-associated structural perturbations and facilitate comprehensive evaluation of protein stability.

Protein aggregates range from nanometer-scale oligomers to micron-sized visible particles, and aggregates of different sizes exhibit distinct immunogenic potentials. Nanometer-scale aggregates (1–100 nm) are already capable of immune recognition and may trigger immune activation. Techniques such as dynamic light scattering and nanoparticle tracking analysis are particularly suitable for detecting these small aggregates and characterizing particle-size distributions. Submicron aggregates (100–1000 nm), which are frequently encountered in pharmaceutical formulations, exhibit enhanced immunogenic potential owing to their larger size and increased immunological visibility. Flow Imaging (FI) is especially effective for monitoring aggregates within this size range, providing detailed information regarding particle count, size distribution, and morphology. Micron-sized and larger aggregates (>1000 nm), which are often visible to the naked eye, carry particularly high immunogenicity risk. Such particles may be assessed through visual inspection, light obscuration methods, and FI-based morphological analysis.

In parallel, AI technologies are emerging as transformative tools for mitigating protein immunogenicity. By leveraging deep-learning algorithms trained on large-scale datasets encompassing protein structures, epitope information, and immune response profiles, AI systems can accurately predict potential antigenic epitopes and design targeted mutational strategies to eliminate T-cell or B-cell epitopes through rational sequence optimization. Generative AI approaches further enable the de novo design of low-immunogenicity protein scaffolds, coupled with biophysical simulations to validate structural stability. More advanced AI-driven protein co-evolution models can simulate immune-evasion mechanisms and iteratively optimize protein surface properties. These computational technologies have already accelerated the discovery and screening of low-immunogenicity candidates in antibody engineering, vaccine development, and therapeutic protein design, thereby driving major advances in precision medicine.

**Figure 5:**
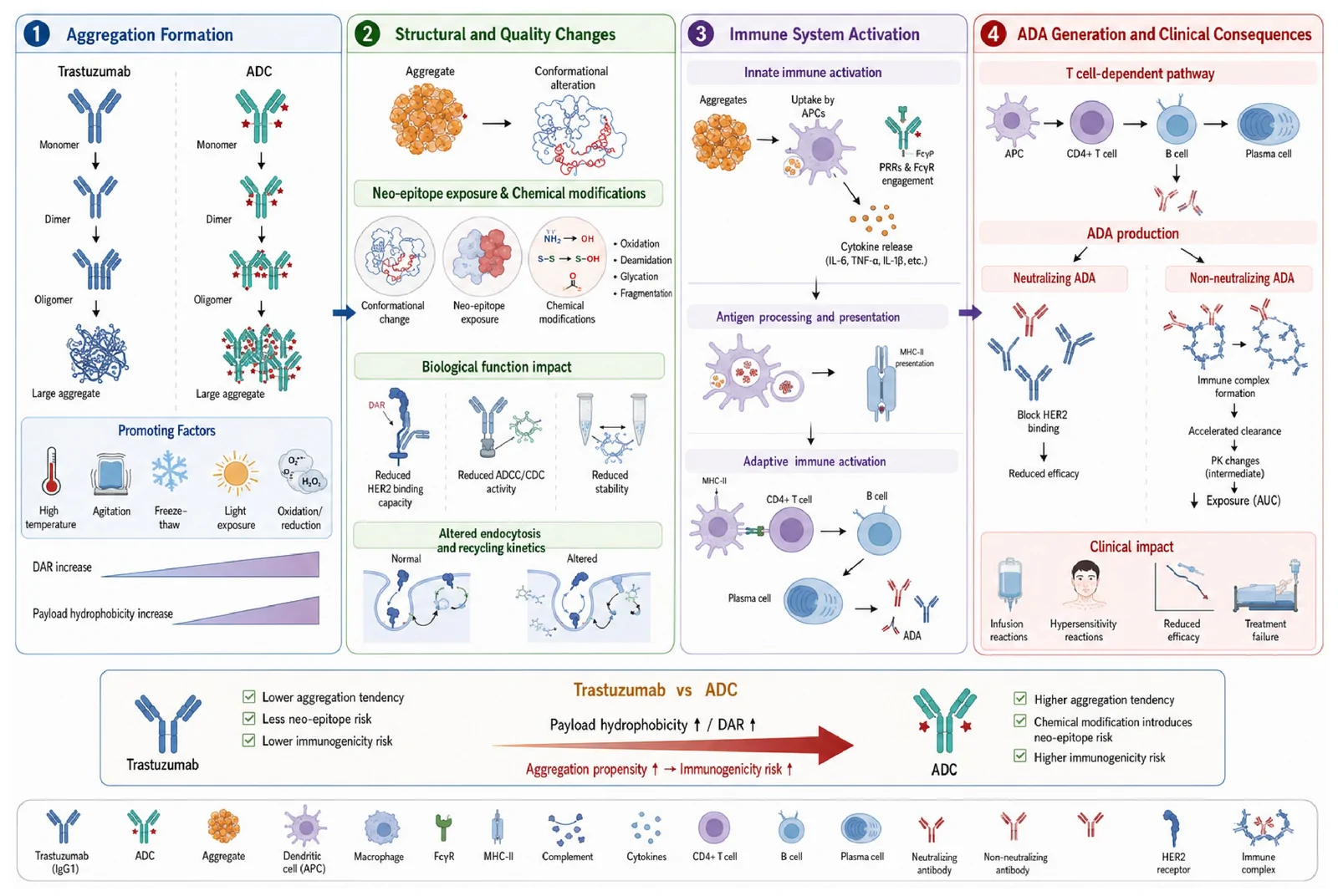
Aggregation-driven immunogenicity in antibody–drug conjugates (ADCs) vs. the naked antibody. Schematic overview of aggregation-induced immunogenicity in ADCs vs. trastuzumab. The process involves the following four key stages: (1) aggregation formation driven by stress factors (e.g., heat, light, high drug-to-antibody ratio [DAR]); (2) structural and quality changes, including conformational alterations and neoepitope exposure, which impair biological function; (3) immune system activation via innate and adaptive pathways, leading to ADA production; and (4) clinical consequences such as reduced efficacy, altered pharmacokinetics, and infusion reactions. The comparison highlights that ADCs, due to their payload hydrophobicity and higher DAR, exhibit greater propensity to aggregation and higher immunogenicity risk than the naked antibody.

Taken together, these analytical and computational strategies enable comprehensive characterization of aggregation-induced structural alterations and aggregation extent, thereby facilitating accurate evaluation of protein stability. Such approaches are of substantial importance for optimizing manufacturing processes, improving formulation design, and controlling storage conditions, ultimately contributing to reduced immunogenicity risk and ensuring the safety and efficacy of therapeutic recombinant biologics. Figure 5 illustrates the mechanism by which aggregation in ADCs—driven by factors such as high drug-to-antibody ratio and payload hydrophobicity—induces immunogenicity through structural alterations and immune activation, ultimately leading to ADA production and adverse clinical outcomes, compared with naked antibodies.

### 7.5. Summary of Strategies

As summarized in Table 3, the strategies discussed throughout this section for reducing protein aggregation and lowering the immunogenicity risk span formulation optimization, molecular engineering, manufacturing-process control, lifecycle management, and emerging computational approaches. Table 3 also provides an assessment of the maturity level of supporting evidence for each strategy, ranging from established clinical evidence to preclinical or research-stage findings. This comprehensive overview underscores the multifaceted nature of aggregation control and highlights the need for an integrated approach across the product lifecycle.

## 8. Conclusions

This review systematically elucidates the mechanisms by which protein aggregation drives the immunogenicity of therapeutic recombinant biologics. Not only do aggregates expose neoepitopes through conformational changes, but they also amplify cytokine release and antigen presentation, thereby activating both innate and adaptive immune pathways to promote ADA generation. Furthermore, the intrinsic immunogenicity of the naked antibody itself must be considered; therapeutics sharing the same target antigen may exhibit similar immunogenic profiles, as the specific target can inherently influence the host’s immune response.

With the emergence of next-generation biologics with complex architectures, such as ADCs, aggregation-induced immunogenicity has become a critical bottleneck for clinical safety. Consequently, aggregate control must be established as a critical quality attribute throughout the product lifecycle. To address this, multidimensional strategies involving structural optimization, precision glycoengineering, and AI-assisted molecular design are essential. In particular, the integration of AI is reshaping the field; by enabling high-throughput prediction and inverse molecular design, AI accelerates the development of low-immunogenicity candidates, marking a paradigm shift from empirical to data-driven protein engineering.

Despite these advances, challenges remain regarding the precise behavior of aggregates in complex clinical environments and the limitations of current detection technologies. Future investigations should focus on elucidating the dynamic immunological consequences of aggregates in patients and developing highly sensitive analytical platforms. By integrating advanced analytics with machine learning, the field can better predict clinical risks. Ultimately, these insights may provide a roadmap for optimizing manufacturing and formulation, reducing immunogenicity-associated risks, and ensuring the safety and efficacy of next-generation therapeutics.

Despite some progress in the study of these mechanisms, clinical translation of the research on immunogenicity related to aggregation remains relatively poor. This field is constrained by numerous overlapping and mutually reinforcing difficulties. Cases of ADA attributable to aggregation are extremely rare in clinical practice, and the causal relationship is extremely difficult to establish due to several reasons: aggregation products are heterogeneous, dynamic, and usually only exist for a short period of time; patients simultaneously receive multiple drug treatments; and there is also immune dysregulation due to underlying diseases and differences in production batches. A conclusive human study requires deliberately administering aggregated and functionally inactive drugs to patients, a design that is ethically unacceptable. Therefore, the design of clinical trials mainly focuses on the efficacy and safety end points rather than on treating aggregation as a controllable variable. Aggregation is rarely prospectively stratified or actively intervened. Moreover, there is currently a lack of standardized, highly sensitive, and validated ADA-detection methods, making it difficult to distinguish ADA reactions driven by aggregation from those caused by other factors related to the product or the patient, especially at low titers and in the presence of circulating drugs. The result is a persistent evidence gap: a phenomenon with mechanistic rationality and reproducible experiments in vitro is difficult to quantitatively and clearly analyze in patients. Therefore, we believe that this field urgently needs to move beyond descriptive clinical observations and shift to integrative, mechanism-based strategies, using carefully designed in vitro cell systems (such as DC maturation, T-cell activation, cytokine release, and BCR cross-linking experiments) and animal models to establish reproducible structure–aggregation–immunogenicity relationships and extrapolate to humans with appropriate caution.

Currently, therapeutic antibodies and recombinant biological agents have benefited a large and continuously growing patient population worldwide. As the range of drug use expands, the absolute number of patients at risk of ADA reactions due to aggregation also increases. Therefore, the industry has a responsibility, and for its own benefit, to actively study the mechanisms of drug aggregation and ADA formation—rather than treating it as an accidental quality attribute—to avoid the occurrence of preventable adverse events. This issue is particularly prominent in oncology: for patients with advanced malignant tumors, ADA-induced severe adverse reactions are often masked or misjudged by disease progression, combined cytotoxic treatments, or multiple immune-related adverse events, and thus, they are not fully identified and reported. However, these patients already bear significant symptom burdens and treatment toxicity; simply ignoring the additional risks and preventable immune burden that may be exacerbated by advanced cancer is unacceptable. Due to the unpredictable consequences of ADA, including loss of efficacy, pharmacokinetic changes, hypersensitivity reactions, and in rare cases, cross-neutralization of endogenous proteins, we call for the development and promotion of more sensitive, standardized, and ADA-detection platforms that can identify aggregation status. It is also important to implement routine longitudinal monitoring in high-risk populations to detect immune-related reactions caused by aggregation, attribute them accurately, and intervene promptly before causing clinical damage.

## Figures and Tables

**Table 1:** Aggregate state and immunogenic potential.

Aggregate Type	Approximate Size	Structural Order	Immunogenic Potential
Monomer (native IgG)	~10 nm	Native, globular	Low
Dimer/soluble oligomer	1–100 nm	Partial, unordered/early β	Moderate—early and recognized
Submicron aggregate (soluble)	100–1000 nm	Amorphous or ordered	High, enhanced immune visibility
Subvisible particle	~1–100 µm	Amorphous (or fibrillar)	High—documented in model systems
Visible particle	>100 µm	Amorphous	Apparent risk; may be cleared
aFibril (amyloid-like)	µm+, ordered	Extensive β-sheet	Notable in model systems

**Table 2:** Analytical methods for aggregate detection and their limitations.

Method	Measures	Size Range	Limitations/Blind Spots
SEC-MALS (size exclusion chromatography with multi-angle light scattering)	Absolute molar mass/radius of gyration (Rg)	~10 nm–1 µm (soluble oligomers)	Limited by column pore size (shear stress); low recovery for very large aggregates; requires soluble species (ICH Q6B Reference)
DLS	Z-avg size/PDI	1 nm–1 µm	Biased to larger species; low resolution; assumes sphericity
NTA	Particle size + concentration	20–1000 nm	Requires dilution; shape dependence
LO (light obscuration)	Particle count/volume equivalent diameter	2–100+ µm	“Blind” to transparent/low refractive index particles (e.g., some protein aggregates); no morphology data (pharmacopeial standard)
FI (flow imaging)	Subvisible particle count + morphology	2–100 µm	Compendial; morphology
Raman microscopy	Chemical composition/secondary structure	~1 µm and above	Low throughput; complex data analysis; diffraction limit restricts small aggregate detection

**Table 3:** Strategies to reduce aggregation and lower immunogenicity, with evidence of maturity.

Strategy	Category	Mechanism	Evidence of Maturity
pH & ionic-strength optimization	Formulation	Minimizing charge-mediated aggregation	Established (clinical)
Sugars/polyols (sucrose, trehalose)	Formulation	Preferential exclusion; conformational stabilization	Established (clinical)
Surfactants (polysorbate)	Formulation	Reduction of interfacial aggregation	Established
PEGylation	Molecular	Steric shielding; reduced immunogenicity	Established
Glycoengineering (glycan/effector)	Molecular	Alteration of aggregation propensity & immune recognition	Emerging
Process control (temp, shear, mixing)	Manufacturing	Prevention of stress-induced aggregation	Standard (GMP)
Cold-chain storage & transport	Lifecycle	Prevention of thermal/stress damage	Standard
Analytical monitoring (SEC, MFI, DLS)	QC/CQA	Detection & control of aggregate levels	Established
AI-assisted inverse design	Molecular	Prediction/design of low-immunogenicity candidates	Preclinical/research

## Data Availability

No new data were created or analyzed in this study. Data sharing is not applicable to this article.

## Abbreviations

The following abbreviations are used in this manuscript:

ADA	Anti-drug antibody
ADC	Antibody–drug conjugate
ADCC	Antibody-dependent cellular cytotoxicity
ADCP	Antibody-dependent cellular phagocytosis
AP-1	Activator protein 1
AI	Artificial intelligence
APC	Antigen-presenting cell
ASC	Apoptosis-associated speck-like protein containing a CARD
AZA	Azathioprine
BCR	B-cell receptor
BSA	Bovine serum albumin
CDR	Complementarity-determining region
CHO	Chinese hamster ovary
CLR	C-type lectin receptor
CQA	Critical Quality Attribute
CRS	Cytokine release syndrome
CTLA-4	Cytotoxic T-lymphocyte-associated protein 4
DAR	Drug-to-antibody ratio
DAMP	Damage-associated molecular pattern
DC	Dendritic cell
DLS	Dynamic light scattering
DLN	Draining lymph node
DM1	Maytansinoid derivative
EPO	Erythropoietin
Fab	Fragment antigen-binding
FcR	Fc receptor
GlcNAc	N-acetylglucosamine
hGH	Human growth hormone
HLA	Human leukocyte antigen
HSA	Human serum albumin
IFN	Interferon
IgG	Immunoglobulin G
IL	Interleukin
IRF	Interferon regulatory factor
IV	Intravenous
LC	Langerhans cell
mAb	Monoclonal antibody
MAPK	Mitogen-activated protein kinase
MAVS	Mitochondrial antiviral-signaling protein
MFI	Micro-flow imaging
MHC	Major histocompatibility complex
MMAE	Monomethyl auristatin E
MoDC	Monocyte-derived dendritic cell
MyD88	Myeloid differentiation primary response 88
MZ	Marginal zone
NAb	Neutralizing antibody
NF-κB	Nuclear factor kappa-light-chain-enhancer of activated B cells
NMR	Nuclear magnetic resonance
NTA	Nanoparticle tracking analysis
OVA	Ovalbumin
PAMP	Pathogen-associated molecular pattern
PD-1	Programmed cell death protein 1
PEG	Polyethylene glycol
pI	Isoelectric point
PRCA	Pure red-cell aplasia
PPR	Pattern recognition receptor
rFVIII	Recombinant human coagulation factor VIII
rhEPO	Recombinant human erythropoietin
rh-Insulin	Recombinant human insulin
RIG-I	Retinoic acid-inducible gene I
ROS	Reactive oxygen species
RPE	Retinal pigment epithelial
SC	Subcutaneous
SVP	Subvisible particle
T-DM1	Trastuzumab emtansine
TBK1	TANK-binding kinase 1
TCR	T-cell receptor
Th	T helper
TLR	Toll-like receptor
Tm	Melting temperature
TNF	Tumor necrosis factor
TRAF	TNF receptor-associated factor
TRIF	TIR domain-containing adapter-inducing interferon-β
UV	Ultraviolet
vc-MMAE	Valine–citrulline-monomethyl auristatin E
VEGF	Vascular endothelial growth factor
VEGFR	Vascular endothelial growth factor receptor
